# Nutritional Management and Biochemical Outcomes during the Immediate Phase after Liver Transplant for Methylmalonic Acidemia

**DOI:** 10.3390/nu12102976

**Published:** 2020-09-29

**Authors:** Casey Siegel, Ronen Arnon, Sander Florman, John Bucuvalas, Kimihiko Oishi

**Affiliations:** 1 Department of Genetics and Genomic Sciences, Icahn School of Medicine at Mount Sinai, New York, NY 10029, USA; casey.siegel@mssm.edu; 2 Department of Pediatrics, Icahn School of Medicine at Mount Sinai, New York, NY 10029, USA; ronen.arnon@mountsinai.org (R.A.); john.bucuvalas@mssm.edu (J.B.); 3 Recanati/Miller Transplantation Institute, Icahn School of Medicine at Mount Sinai, New York, NY 10029, USA; Sander.Florman@mountsinai.org

**Keywords:** Methylmalonic acidemia, MMA, liver transplant, nutrition

## Abstract

Methylmalonic acidemia (MMA) is caused by a deficiency of methyl-malonyl-CoA mutase. It is a multisystemic condition with poor clinical outcomes characterized by frequent metabolic decompensation with acidosis, hyperammonemia and encephalopathy. Restriction of intact protein and supplementation with amino acid-based formula play an important role in its management. Recently, liver transplant (LT) became a treatment option for MMA patients. However, there has been no current consensus on the post-operative nutrition management for MMA patients undergoing transplant, particularly during the initial phase of recovery period with catabolic stressors. We performed a retrospective analysis of clinical and nutritional management as well as biochemical profiles before and after LT in five patients with MMA. Through this study, we observed significant improvement of MMA-associated metabolites after LT. MMA patients were able to tolerate increased intact protein intake post-operatively. At least 1–1.5 g/kg/day of total protein during the acute phase after transplant may be tolerated without worsening of the metabolite levels. This information provides a guide in how to nutritionally manage MMA after LT.

## 1. Introduction

Methylmalonic acidemia (MMA) (OMIM: 251000) is an organic acidemia caused by pathogenic variants in the *MMUT* gene encoding the enzyme, methyl-malonyl-CoA mutase, which is important for the metabolism of valine, methionine, isoleucine, threonine and odd-chain fatty acids [1]. The enzymatic defect causes accumulations of methylmalonic acid in the body, resulting in multisystemic disease. It can present as lethargy, vomiting, metabolic acidosis, hyperammonemia and encephalopathy during the newborn period and can result in coma or death if untreated [2]. Long-term complications include developmental delay and intellectual disability, hypotonia, cardiomyopathy, pancreatic insufficiency, osteopenia and renal dysfunction [3]. Because of the enzymatic defect, MMA patients are very sensitive to protein loads and an excessive amount of protein intake or increased catabolism can trigger a higher accumulation of methylmalonic acid leading to metabolic acidosis [3]. Dietary protein restriction with precursor-free amino acid (PFAA) formulas, which are methionine- and valine-free with low isoleucine and threonine, and carnitine supplementation play an important role in its medical management. Despite currently available medical and nutritional interventions, patients with MMA remain at risk for metabolic decompensations leading to poor neurological outcomes.

Patients with MMA have feeding difficulties for a variety of reasons, such as poor appetite, developmental delays, and nausea, as well as the decreased palatability of metabolic formulas [4,5]. Often these patients require enteral nutrition support via nasogastric (NG) or gastrostomy tube (GT) to meet their nutrition needs and prevent catabolism. While some patients use those tube feedings only for administration of metabolic formula and/or medication, there are patients who are 100% dependent on NG or GT to meet their nutritional requirement. To achieve optimal management, close monitoring of biochemical markers and nutritional balance between intact protein and amino acid-based medical formula by specialized physicians and dietitians is essential [2,6]. 

Recently, liver transplant (LT) became a treatment option for MMA. While it is considered non-curative, it can improve metabolic control, decrease frequency of decompensations and hospitalizations, and ease diet restrictions [7,8,9,10]. However, the process of organ transplant is a significant metabolic stressor that includes a long period of fasting, invasive surgical procedures, and corticosteroid use. It has been shown that protein requirement and catabolism are significantly increased during the immediate post-operative phase in all liver transplanted patients [11,12,13]. Since the metabolic defect of the organs other than the graft liver is unchanged, it is possible that the systemic production of methylmalonic acid and other toxic metabolites is increased when patients are exposed to overwhelming stressors associated with LT, even with a functioning graft liver. Therefore, careful perioperative nutritional support is imperative for successful post-operative wound healing and recovery, graft functioning, and metabolic control of MMA. 

Despite many successful LTs, the information regarding the perioperative nutritional support has not been well described and the ideal nutritional management during LT post-operative remains unclear. In this single-center study, our goal was to investigate the effects of LT on biochemical profiles and review the nutritional support provided in patients with MMA who received LT at our center.

## 2. Patients and Methods 

A single-center retrospective analysis of patients who underwent LT for MMA at Mount Sinai Hospital from September 2014 to August 2019 was performed. Electronic chart review to collect clinical information, nutritional management history, and biochemical test results was carried out. For the purpose of review, perioperative nutritional profile, medical and nutritional management and biochemical profiles before and after LT were analyzed. Detailed nutritional management information during the first two weeks after LT was reviewed. Biochemical test values from one year prior to LT were collected and summarized with median and interquartile range (IQR). Wilcoxon rank sum test was used to analyze the changes in biochemical parameters during the one year period before transplant and the period after transplant. A *p*-value of <0.05 was considered statistically significant. STATA 14.2 (StataCorp, College Station, TX, USA) statistical software was used for statistical analyses. This study was approved by the Institutional Review Board of the Icahn School of Medicine at Mount Sinai. 

All patients were confirmed to be metabolically stable without acidosis before transplant and received 10% dextrose intravenous fluid at 1.5 times their maintenance fluid rates during the preoperative fasting period. Transplant procedures were performed under general anesthesia. Midazolam, propofol and fentanyl as well as muscle relaxants including vecurinium or rocurinium were used. Electrolytes and acid base balance were carefully monitored during the procedure. Immediately postoperatively, patients were transferred to intensive care units for close monitoring. Fentanyl and dexmedetomidine were used for analgesia and sedation. Corticosteroids, tacrolimus, and mycophenolate mofetil were used for immunosuppression from the postoperative day (POD) #1. For pediatric patients, methylprednisolone was initiated with a starting dose of 2.5 mg/kg (for patients weighing <20 kg) or 50 mg (20 kg or more) four times a day and the dose was tapered to 0.5 mg/kg and 10 mg, respectively, over five days. For an adult patient who received combined liver/kidney transplant, methylprednisolone was initiated with a starting dose of 125 mg/m^2^/day (80 mg two times a day) on POD#1 and the dose was tapered to 20 mg two times a day over four days. Methylprednisolone therapy was followed by oral prednisolone with a daily dose of 3 mg (Age < 5 years), or 5 mg (Age 5–12 years). For adult patients, this was followed by a daily dose of 20 mg of oral prednisone and the dose was tapered slowly over weeks. Mycophenolate mofetil was discontinued three months after isolated LT. Oral corticosteroid therapy was continued until six months after LT in patients who received isolated LT. The lengths of treatment with mycophenolate mofetil and low dose oral prednisolone were adjusted and were longer in patients who underwent combined liver/kidney transplant. All patients continued on tacrolimus without discontinuation. 

## 3. Results

### 3.1. Patient Characteristics

Five patients with MMA (four females and one male) received liver or liver/kidney combined transplant during the five-year period (Table 1). Except for Patient #1, all patients were identified by newborn screening. Patient #4 and #5 are siblings. Diagnosis was confirmed by biochemical tests and/or genetic sequencing of the *MMUT* gene. They are all still alive and their current age range is 3 to 28 years. The range of their ages at the transplant was between 35 months and 25 years old. All patients received LT because of difficulties of metabolic control and four out of five patients received combined liver and kidney transplant because of renal insufficiency. Patient #2 initially received only LT, but had deceased donor kidney transplant 12 months after LT due to worsening kidney function, likely triggered by tacrolimus associated kidney injury in the face of preexisting subclinical kidney disease from MMA. All five patients needed GT feeding due to a history of poor feeding. Mild to severe developmental delay was observed in three patients. Patient #3 had mild truncal hypotonia that was improved by physical therapy. On the other hand, Patient #4 and #5 had moderate/severe developmental delay in multiple developmental aspects including gross and fine motor, cognitive, and language functions, likely to be due to the high level of exposure to MMA and frequent episodes of metabolic decompensation with acidosis. There were no intraoperative complications for all patients. Cold/warm graft liver ischemic times during the procedure were in the range of 5–8 h (median: 6.6 h) and 25–34 min (median: 30 min). All patients except for Patient #2 received 5% albumin solution during the transplant procedure to maintain oncotic pressure, without complications. None of the patients received fresh frozen plasma during the procedure. During the acute phase after transplant, three patients (Patients #3, #4, and #5) had respiratory complications including respiratory distress and viral infection. Patient #5 had renal vein thrombosis and the graft kidney was removed 30 days after transplant. During the initial six months post-LT period, there were no acute graft liver complications including acute rejection, hepatic artery thrombosis, or significant hepatic synthetic dysfunction for the other patients. No signs and symptoms suggesting metabolic acidosis were observed. However, there were some long-term complications observed in the patients. Patient #1 had a new onset of tacrolimus-associated seizures at two months after the transplant that was controlled by anti-seizure medications and resolved one year after transplant. Patient #2 had a kidney transplant due to tacrolimus-induced nephrotoxicity (see above). Patient #3 had BK virus infection and has been managed with modifications of immunosuppression and intravenous immunoglobulin (IVIG) administrations. Patient #4 had an episode of mild rejection that was completely resolved with a short course of corticosteroid. This patient developed post-transplant lymphoproliferative disease (PTLD) one year after transplant that was treated with decreased immunosuppression and rituximab. Patient #5 had episodes of hyponatremia, viral gastroenteritis, and adenovirus infection between 6–12 months after transplant and these were treated by supportive management. Patients’ information including graft outcome and complications are summarized in Table 1.

### 3.2. Biochemical Profile

**Figure 1:**
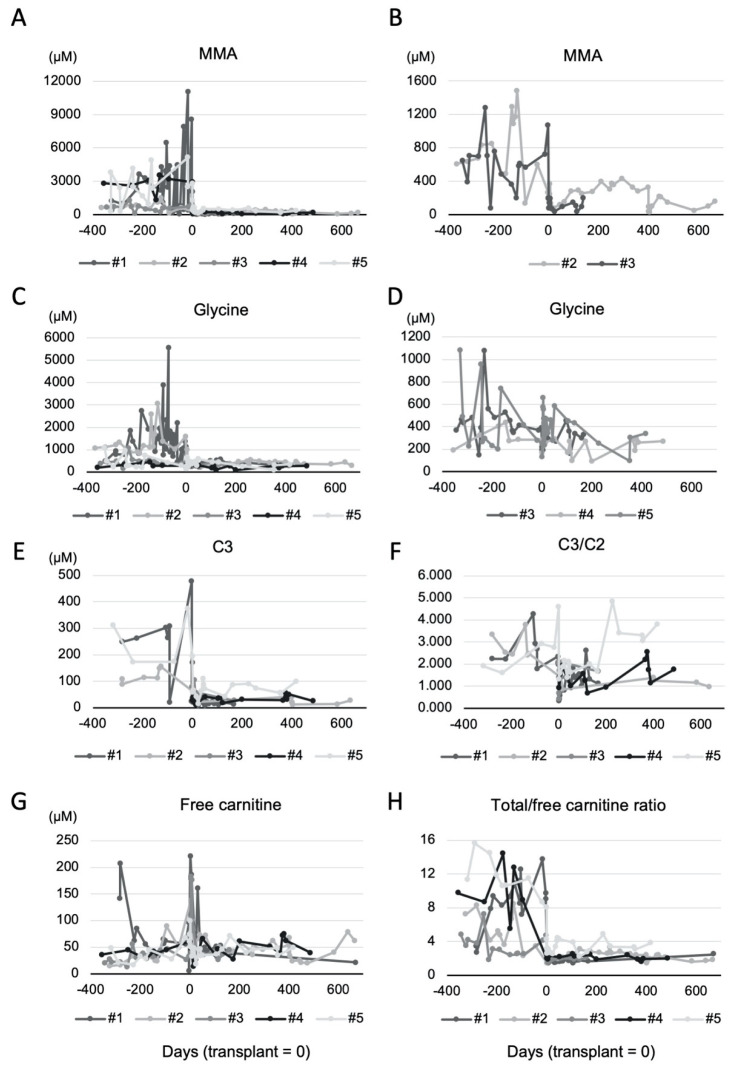
Biochemical profiles of patients six months before and after liver transplant. (**A**,**B**); plasma MMA level, (**C**,**D**); plasma glycine level, (**E**); plasma C3 level, (**F**); plasma C3/C2 ratio, (**G**); plasma free carnitine level, (**H**); plasma total/free carnitine ratio. (**B**,**D**) graphs represent values of patients who had lower MMA or glycine levels compared to the other patients, respectively. 0 of *x*-axis represents the day of transplant. Numbers of *x*-axis represent patients’ ID. MMA; methylmalonic acid, C3; propionyl-carnitine, C2; acetyl-carnitine.

**Figure 2:**
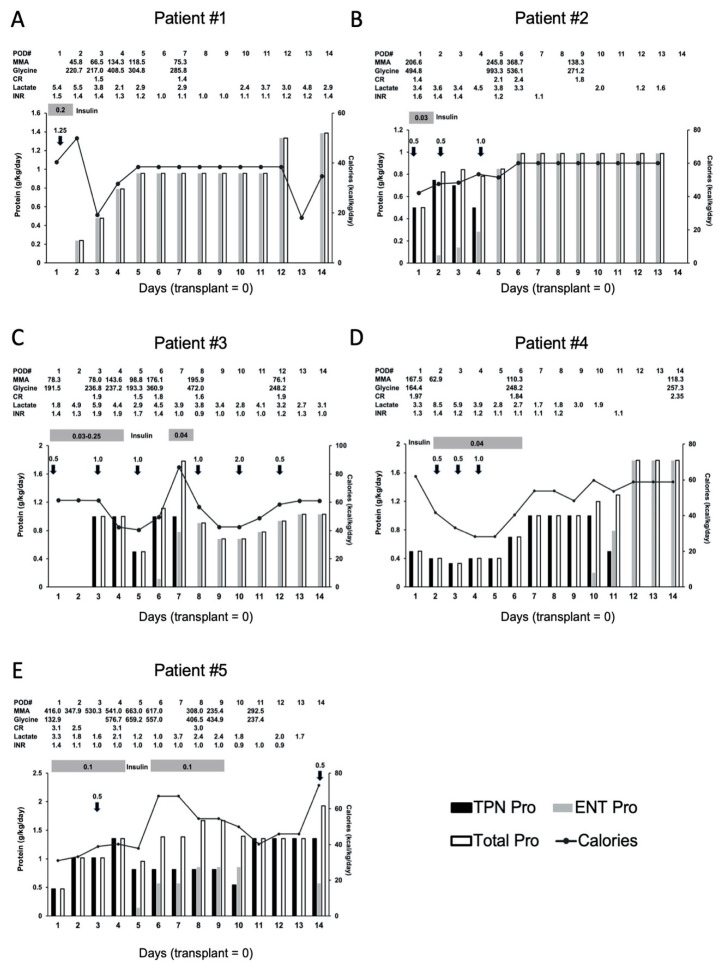
Nutritional management 2 weeks after liver transplant of (**A**) Patient #1, (**B**) Patient #2, (**C**) Patient #3, (**D**) Patient #4, and (**E**) Patient #5. Daily protein (vertical bars) and calorie (black line) intakes are shown. Black vertical bars: protein intake from total parenteral nutrition (TPN) (g/kg/day); grey vertical bars; protein intake from enteral feeding (ENT) (g/kg/day); white vertical bars: total protein intake (Total) (g/kg/day); black arrows: albumin infusion with dose (g/kg/dose); grey horizontal bars: insulin infusion with dose (units/kg/h). Normal ranges and units; plasma MMA = 0–0.4 μM, glycine = 127–341 μM, lactate = 0.5–2.2 mM. MMA; methylmalonic acid, CR; plasma total/free carnitine ratio.

Biochemical markers including plasma methylmalonic acid, propionyl-carnitine (C3), methylmalonic/succinyl-carnitine (C4DC), C3/C2 (acetyl-carnitine) ratio, plasma carnitine profile, and plasma amino acids from one year before LT were reviewed and summarized in Table 2. As shown in Figure 1A,B, methylmalonic acid levels were significantly elevated in all patients and these values decreased immediately after transplant. However, even with this significant change, methylmalonic acid levels have never normalized. During the first two weeks after LT, the diminished methylmalonic acid levels were stably maintained even with multiple catabolic stressors (Figure 2). Pre-transplant glycine level was remarkably increased in two patients (Patient #1 and #2) and minimally elevated in one patient (Patient #3) preoperatively. After LT, the abnormally elevated glycine was also immediately normalized (Figure 1C,D). While not being as dramatic compared to the changes of methylmalonic acid and glycine, plasma C3, C4DC, C3/C2 acylcarnitine ratio were also improved with LT. Overall, the medians of free carnitine level before transplant were in the normal reference in all patients because of carnitine supplementation. The median of free carnitine of all patients after transplant was mildly increased compared to the pre-transplant period (Table 2). Carnitine profile was influenced by the route and dose of carnitine supplementation, particularly by its intravenous administration. As shown in Figure 1G, there were noteworthy elevations of free carnitine between post-operative days 0–4, which was a reflection of intravenous carnitine administrations while oral supplementation was not available. Therefore, we calculated the ratio between total and free carnitine concentrations to indirectly evaluate the degree of disease-specific metabolite load. As predicted, this carnitine ratio was significantly decreased in all patients with transplant, suggesting the reduced amounts of accumulated metabolites such as 3-hydroxypropionic acid, methyl-citric acid and/or methylmalonic acid in plasma (Table 2, Figure 1G).

### 3.3. Post-Transplant Nutritional Parameters and Interventions

Nutritional interventions and outcomes six months before and after transplant are summarized in Table 3 and Figure 2. During the pre-operative fasting period, all patients received 10% dextrose intravenous fluid via peripheral venous catheters at 1.5 times maintenance fluid rates. For all patients except for Patient #1, total parenteral nutrition (TPN) with 5–20% dextrose was used because of the need for respiratory support, intestinal dysmotility, or feeding intolerance. Patient #1 did not receive TPN since she was extubated earlier, she was clinically stable and early advancement of the enteral feeding to her goal rate was expected. Similarly, Patient #2 was extubated early and enteral feeding was started immediately after that, but his respiratory rate was mildly elevated after extubating. Therefore, his feeding rate was advanced slowly while giving him TPN. TPN contains mixed amino acid solutions without restricting the precursor amino acids. It was initiated between POD #1 and #3 and the duration of TPN administration was 3 to 15 days. Of the four patients that received TPN, three were started on POD#1. TPN was started with an initial protein dose of 0.5 g/kg/day and this was gradually increased to 1.0–1.5 g/kg/day. All patients received 0.02–0.25 units/kg/h of regular insulin for hyperglycemia post-operatively. At least one dose of albumin infusion was given to all as a part of post-operative management. Enteral feeding was initiated as early as possible, with four out of five patients initiated within the first week. However, the other patient required a much longer time due to respiratory complications and feeding intolerance with vomiting (Table 1 and Table 3, Figure 2). TPN was weaned once enteral feeds reached 50% of goal. Enteral feeds only needed to take place for more than a few hours for Patient #4 and they were restarted within four days. TPN and enteral feeds were adjusted daily based on tolerance, to provide goal protein and calories. Total protein from nutrition support was increased gradually while metabolites remained decreased from baseline. During the first two postoperative weeks, plasma methylmalonic acid levels continued to be much lower than preoperative values and plasma glycine levels were always in the normal range (Figure 2). Lactate levels were lower overall than those of preoperative values but there were occasional elevations during the first two weeks. However, they were not associated with worsening MMA-related metabolites or nutritional interventions (Figure 2). 

After transitioning to enteral feeding, the total protein intake goal from postoperative nutrition support was increased and individualized based on guidelines for critical illness, while monitoring biochemical parameters for MMA [8]. All patients were able to tolerate a higher load of intact protein while maintaining improved biochemical parameters. Two patients were able to achieve protein goal within the first week after transplant and the remaining three patients achieved protein goal within two weeks. Patient #3 met the initial inpatient protein goal and this was able to increase further during outpatient follow up. Prior to transplant, four out of five patients required some amino acid-based PFAA formula to meet protein requirements. At six months post-transplant, these patients no longer required PFAA formula and all protein was from an intact source (Table 3). Patient #2 tolerated a higher amount of intact protein immediately following liver transplant, but protein eventually needed to be reduced due to decline in kidney function. Following kidney transplant, his intact protein was again increased to greater than pre-transplant tolerance. Since all patients became able to meet their protein goals fairly early and these were maintained, overall there was no significant change in their nutrition at the time of this chart review after the six months post-transplant period. 

For the four pediatric patients who were still growing at time of LT, growth was not significantly affected at six months post-transplant (Table 3). Patient #3 showed a minor increase in height/age z-score at 6 months which continued at one year post-transplant with improvement in z-score by 1.0. Patient #1, who was an adult at time of transplant, showed improvement in body mass index (BMI). Prior to transplant her BMI was considered underweight and six months post-transplant her BMI was considered normal. 

All patients received oral carnitine supplement pre-operatively to maintain their free carnitine level. Until they restarted enteral feeding, intravenous supplementation of carnitine at the same daily preoperative dose was given. Because of the intravenous administration, some patients showed a significant temporary surge of plasma free carnitine level (Figure 1 and Table 2). Carnitine dose was adjusted to keep the plasma free carnitine level within the normal reference range. Compared with the pre-operative doses, their oral carnitine dose was decreased for all patients and none of them discontinued the supplementation. 

## 4. Discussion

MMA is a life-threatening inborn error of the metabolism with devastating multisystemic complications including metabolic acidosis, neurological impairments, strokes, developmental delay, and renal insufficiency [1]. Recently, medical management with dietary protein restriction has been improved; however, the risk of poor neurological outcomes and progressive renal dysfunction can be inevitable particularly for severe MMA cases. During the past decade, an increasing number of MMA patients received liver or combined liver/kidney transplant and favorable biochemical outcomes have been reported [8,14,15,16,17]. Consistent with the previously reported transplanted cases, our patients showed significant improvements in their plasma methylmalonic acid level after LT. Similarly, the significant decreases of plasma glycine level and total/free carnitine ratio were observed, suggesting improvement of the MMA-associated metabolite load in the body. Overall, biochemical profiles showed the expected improvement with good liver graft functions, at least for the observation period after transplant, the time frame of the biochemical observation analysis of this study. Despite the successful LT, one patient who did not have combined liver/kidney transplant experienced worsening renal function after LT. It was possible that he had subclinical renal insufficiency due to his underlying MMA even though his glomerular filtration rate (GFR) was not reduced without significant elevations of creatinine and cystatin C. It was suggested that a nephrotoxic immunosuppressant, tacrolimus is likely to have triggered worsening kidney injury in the face of preexisting subclinical kidney disease from MMA. He eventually received a deceased donor kidney transplant a year after the liver transplant. The ideal timing of combined liver/kidney transplant is still unclear and decision making is difficult [18], but a combined liver/kidney transplant may be considered for patients with MMA even when there is no significant reduction of renal function. It has been proposed that early isolated LT should be considered ideally within the first year of life in all patients with severe forms of MMA such as those who have neonatal-onset, cobalamin-unresponsive MMA, in order to prevent irreversible neurological complications [17,19,20]. Additionally, it is suggested that early LT can be beneficial to preserve renal function and avoid or at least delay the indication of kidney transplant. More longitudinal studies including long-term follow up data analysis of patients would lead to firm guidelines surrounding liver +/− kidney transplant for MMA.

Unlike other metabolic conditions such as urea cycle disorders, liver transplant is not a curative treatment for MMA [9,10]. Although liver transplant ameliorates the disease, patients with MMA continue to produce methylmalonic acid and other metabolites even with a functioning graft liver [17]. With a potential risk of renal dysfunction, careful postoperative nutritional and biochemical monitoring are essential. Optimal protein intake depends on each individual’s metabolic needs and biochemical profiles, and post-operative specific nutritional management guidelines have not been well established, including nutritional management during the immediate phase after transplant procedures. Especially, protein catabolism is known to be remarkably increased during the immediate phase after transplant [13]. This can be due to the mixture of surgical stress, prolonged fasting, and steroid use. In general, overwhelming catabolism causing high systemic protein loads is a risk for metabolic decompensation in MMA with a worsening methylmalonic acid level [2,3]. In order to understand protein intake, along with total caloric intake and other nutritional factors including albumin infusion, we reviewed detailed nutritional management for our patients as shown in Figure 2. Even in this stressful setting, our patients tolerated the stress of the transplant procedures as well as TPN with precursor amino acids (up to 1–1.5 g/kg/day of total protein) and albumin infusions (0.5–1.25 g/kg/day) combined with regular insulin infusion to promote anabolism (Table 3 and Figure 2). During this period, total daily protein intake occasionally exceeded the amount of protein that they had taken before transplant. However, the levels of their methylmalonic acid, glycine, and total/free carnitine ratio remained stably lower than their preoperative values (Figure 1 and Figure 2, Table 1). There were occasional episodes of lactate elevation during the first two weeks, but they did not seem correlated with the amount of protein intake and other biochemical markers. This suggests that lactate value is not useful to directly monitor the requirement of protein intake; rather it reflects other systemic conditions such as hypoperfusion. These observations suggest that patients with MMA can tolerate at least 1–1.5 g/kg/day of total protein from TPN and albumin infusions during the acute phase after transplant without worsening of the MMA-associated metabolite levels as long as a graft liver shows improving hepatic synthetic function. The total protein dose included in the TPN that our patient received was still lower than that from a previously published article with guidelines for nutritional management for liver transplant patients [21]. In fact, it is still unclear how much protein patients with MMA can tolerate. However, a dose of 1–1.5 g/kg/day of total protein during this period seems acceptable given the good liver graft functions and recovery of our patients.

After the immediate phase, our patients were successfully transitioned to enteral feeding within several days of surgery except for two patients who took 10–14 days to reestablish enteral feeding due to the requirement of prolonged respiratory support and feeding intolerance (Table 3). Since then, they were able to reach their target protein intake at least within two weeks. Total protein intake was similar between pre- and post-transplant six-month periods. However, the ratio of intact protein in the diet was changed after transplant. Except for patient #3, all patients received substantial amounts of PFAA accounting for 20–30% of total daily protein intake. After transplant, all protein intake was from intact protein sources for all five patients. This observation replicates the findings of previously reported studies, indicating that patients with MMA can be nutritionally managed with dietary sources of intact protein only after liver transplant [16]. The six months post-operative time frame is not sufficient to evaluate the effect of nutritional management on growth particularly of pediatric patients. The nutritional management at least did not have a significant negative impact on their linear growth during this period. It is possible that improved growth for the patient without progressive kidney disease may be achievable. According to previous studies with a longitudinal outcome analysis, improved linear growth can be seen [8].

As previously described, MMA patients continue to produce a substantial amount of methylmalonic acid even with functioning graft liver [16,17]. This was similar for our patients, and their methylmalonic acid levels were still much higher than the normal reference range. Due to the nephrotoxicity of methylmalonic acid, renal disease can continue to progress even after liver transplant, and plasma methylmalonic acid could rise as GFR declines [22]. In this setting, PFAA formula may need to be potentially reintroduced to reduce the methylmalonic acid level. Thus far, information on this aspect is limited, and additional research is needed for clarification. Nevertheless, close monitoring of nutritional and biochemical parameters along with kidney function is necessary for MMA management. 

Carnitine is an essential nutrient in energy production through transporting long-chain fatty acids into mitochondria and for removing various metabolites from cells [23]. In MMA, carnitine supplementation for replacing the carnitine pool in the body is recommended to support the conjugation and excretion of C3 as a part of standard nutritional management [1]. The dosing of carnitine supplementation for our patients was adjusted according to their plasma free carnitine concentrations and our observation was consistent with previously reported findings that carnitine continues to be required after liver transplant [16]. Our patients did not require the same or a higher dose of carnitine compared to the pre-transplant dose and 20–80% of the original dose for each individual seems sufficient to maintain the appropriate plasma concentration of carnitine (Table 3). Of interest, there was an unusual surge of plasma carnitine observed post-operatively for some patients (Figure 1). This was normalized as soon as carnitine supplementation was switched to the oral route. Previous research suggested that a metabolite, trimethylamine N-oxide metabolized from carnitine by intestinal bacteria might increase the risk of cardiovascular events [24]. Although the duration of the exposure to high concentrations of plasma carnitine is limited, adjustment of the dose of intravenous carnitine supplementation needs to be considered. 

There are some limitations relating to this study. These include the small sample size in a single-center setting with only five patients enrolled. Additionally, the study period was limited by retrospective chart reviews. The findings, particularly about protein tolerance after LT, need to be carefully interpreted and used for future MMA patients who will undergo LT. Unfortunately, it is difficult to conduct clinical studies with a large sample size on rare disease such as MMA [25]. Thus, a future longitudinal prospective study with a larger sample size is necessary to investigate and to reproduce the effects of LT for MMA. Nonetheless, our study at least gave us some insights into the biochemical and nutritional efficacies of LT for MMA.

In summary, we described the clinical and biochemical outcome of LT and reviewed nutritional management during the early phase of post-transplant period in our patients with MMA. It was shown that patients with MMA were able to tolerate at least 1–1.5 kg/day of protein loads by TPN and albumin infusions after transplant of liver or liver/kidney despite the significant catabolic status from surgical procedures and steroid administration without worsening of the MMA-associated metabolite levels. Additionally, improvements of nutritional status were achieved after LT. Nutritional support is important even after LT and our report will be helpful in guiding postoperative nutritional management for patients with MMA, but further studies are necessary to achieve more optimal nutritional management to improve quality of life.

## Figures and Tables

**Table 1:** Patient Demographics and Clinical Information.

Patient ID	#1	#2	#3	#4	#5
Gender	Female	Male	Female	Female	Female
*MMUT* nucleotide change (NM_000255.3)	c.[983T>C]; [2150G>T]	c.[322C>T]; [1218del]	c.[983T>C]; [1022dup]	N/A	N/A
Protein change	p.[L328P]; [G717V]	p.[R108C]; [N407Tfs*24]	p.[L328P]; [N341Kfs*20]	N/A	N/A
Age of Transplant	25 y 11 m	5 y 1 m	2 y 9 m	5 y 2 m	4 y 7 m
Weight at LT	50 kg	26.3 kg	14.8 kg	18.9 kg	17.7 kg
Current age	28 y	7 y	3 y	7 y	6 y
Pre-transplant complications	Renal failure on dialysis	No	Renal insufficiency and mild hypotonia	Renal insufficiency and developmental delay	Renal insufficiency and developmental delay
Gastrostomy feeds required	Yes	Yes	Yes	Yes	Yes
Type of Transplant	Deceased Donor Liver/Kidney Combined	Deceased Donor Liver	Deceased Donor Liver/Kidney Combined	Deceased Donor Liver/Kidney Combined	Deceased Donor Liver/Kidney Combined
Graft type	Whole liver/kidney	Whole liver	Whole liver/kidney	Whole liver/kidney	Whole liver/kidney
Cold/Warm Graft Liver Ischemic Time	5 h /30 min	8 h /31 min	6.6 h /34 min	5.5 h /25 min	7 h /25 min
Intra-operative FFP use	No	No	No	No	No
Intra-operative albumin use (Dose of 5% of albumin)	Yes (1500 mL)	No	Yes (250 mL)	Yes (250 mL)	Yes (600 mL)
Post-operative immediate complications	No	No	Fever and respiratory distress, transient abnormal movement	Rhinovirus/enterovirus infection and atelectasis	Tachypnea requiring respiratory support, graft renal vein thrombosis
Follow up period after LT for this study	19 months	11 months	6 months	16 months	14 months
Graft outcome	Functional	Renal insufficiency and kidney transplant 12 m after LT	Functional	Functional	Liver: functional Kidney: graft failure—explanted on POD#30
Complications †	Tacrolimus-associated seizures resolved within 6 months after transplant, Weissella confusa bacteremia, C. Diff infection	Tacrolimus nephrotoxicity. C. Diff infection -resolved with fecal transplant	BK virus infection	PTLD, one episode of mild rejection	Hyponatremia, viral gastroenteritis, adenovirus infection

N/A: not assessed; LT: liver transplant; C. Diff: Clostridium difficile; FFP: fresh frozen plasma; POD: post-operative day; PTLD: post-transplant lymphoproliferative disease; y: years; m: months. * (asterisk) means translation termination (stop) codon to describe a type of genetic variant. †: post-operative complications including vascular/biliary problems.

**Table 2:** Biochemical Outcome.

	Median Value (IQR)							*p* Value *
Patient ID		#1	#2	#3	#4	#5	All Patients	
Plasma MMA (μM)	Pre-LT	2177.1 (428.2–4115.3)	657.2 (492.0–1091.8)	599.7 (366.0–709.0)	2923.9 (2616.7–3197.4)	2453.0 (1266.0–3796.3)	1082.8 (517.4–2920.6)	
	Post-LT	75.8 (51.9–107.0)	217.7 (102.7–305.7)	97.9 (76.1–147.9)	119.3 (65.1–176.4)	347.9 (262.6–514.3)	140.7 (78.3–276.3)	<0.001
Plasma C3 acylcarnitine (μM)	Pre-LT	255.6 (73.0–302.4)	113.6 (108.8–145.0)	80.3 (N/A)	205.6 (N/A)	195.5 (173.7–310.9)	172.2 (108.8–264.5)	
	Post-LT	22.0 (15.7–31.2)	21.5 (17.2–34.6)	57.5 (36.1–59.8)	31.2 (25.8–41.0)	66.3 (53.9–89.1)	31.5 (21.2–57.1)	<0.001
Plasma C4DC acylcarnitine (μM)	Pre-LT	3.4 (1.9–6.1)	1.5 (1.4–1.6)	0.8 (N/A)	4.0 (N/A)	2.7 (2.5–3.7)	2.5 (1.4–3.8)	
	Post-LT	0.7 (0.5–1.0)	2.3 (1.1–3.9)	0.9 (0.6–1.1)	1.0 (0.8–1.1)	2.4 (1.8–2.9)	1.0 (0.8–1.8)	<0.001
C3/C2	Pre-LT	2.2 (2.0–2.7)	2.5 (2.4–3.3)	2.1 (N/A)	2.1 (N/A)	2.8 (1.9–2.9)	2.4 (2.0–2.9)	
	Post-LT	1.2 (0.8–1.5)	1.3 (1.1–1.4)	1.6 (1.4–1.7)	1.4 (1.0–1.8)	1.8 (1.4–2.1)	1.4 (1.1–1.7)	<0.001
Plasma free carnitine (μM)	Pre-LT	61.3 (55.8–85.3)	33.0 (23.9–36.1)	31.4 (22.4–39.4)	40.3 (35.9–44.7)	38.8 (27.9–60.6)	38.8 (27.9–56.1)	
	Post-LT	45.8 (39.0–53.5)	51.3 (40.3–68.2)	44.8 (36.2–85.6)	49.7 (38.1–62.4)	45.9 (34.7–71.2)	47.3 (38.1–65.9)	<0.005
Total/free carnitine ratio	Pre-LT	9.1 (7.9–10.8)	5.0 (3.6–7.2)	3.3 (2.5–4.2)	9.3 (8.7–12.8)	11.0 (8.6–11.8)	7.5 (3.8–9.7)	
	Post-LT	1.7 (1.6–1.8)	2.2 (1.8–2.5)	1.9 (1.8–2.0)	2.0 (1.9–2.4)	3.2 (2.7–3.9)	2.0 (1.8–2.5)	<0.001
Plasma glycine (μM)	Pre-LT	1219.0 (803.4–1878.8)	1154.3 (1042.0–1485.3)	412.3 (367.6–481.5)	286.6 (272.3–405.8)	286.5 (243.0–615.9)	856.5 (399.2–1348.5)	
	Post-LT	338.0 (267.4–413.2)	373.5 (325.5–465.4)	296.6 (237.0–361.4)	258.5 (170.6–271.9)	420.7 (277.4–467.4)	338.3 (263.6–436.7)	<0.001
Plasma glutamine (μM)	Pre-LT	341.3 (253.7–406.1)	474.4 (378.6–571.2)	378.5 (318.3–404.3)	371.5 (362.9–441.7)	314.5 (290.0–444.9)	370.9 (304.5–436.7)	
	Post-LT	567.6 (505.7–667.4)	533.3 (446.0–586.7)	402.1 (304.8–444.6)	408.5 (254.5–524.6)	506.4 (354.0–645.5)	504.0 (395.4–585.0)	<0.001

* *p* value was calculated by Wilcoxon rank sum test for all patients between pre- and post-liver transplant. Normal range; plasma MMA = 0–0.4 μM, plasma C3 acylcarnitine = 0–1.27 μM, C4DC acylcarnitine = 0–0.15 μM, C3/C2 < 0.1, free carnitine = 24–63 μM, glycine = 127–341 μM, and glutamine = 254–823 μM. LT; liver transplant, IQR; interquartile range, MMA; methylmalonic acid; N/A; not available.

**Table 3:** Post-operative nutritional interventions and outcome for the six months duration before and after liver transplant.

Patient ID		#1	#2	#3	#4	#5
Total protein intake (g/day)	Pre-LT	30.0	24.9	13.8	20.1	15.9
	Post-LT	42.0	23.3	22.5	30.7	18.0
Total protein intake (g/kg/day)	Pre-LT	0.70	1.03	0.98	1.10	1.00
	Post-LT	0.81	0.81	1.36	1.59	0.99
Intact protein (% total daily protein intake)	Pre-LT	67.22	87.74	100.00	70.19	71.72
	Post-LT	100.00	100.00	100.00	100.00	100.00
Linear growth with height z-score	Pre-LT	N/A	0.81	−0.85	−2.33	−2.22
	Post-LT	N/A	0.82	−0.12	−2.85	−2.28
Weight z-score (except for #1)	Pre-LT	45.7 kg	2.21	−0.80	−2.33	0.43
	Post-LT	51.7 kg	1.46	1.1	−2.85	0.18
Carnitine dose (mg/kg/day)	Pre-LT	53.3	136.9	20.3	48.4	56.5
	Post-LT	11.6	51.8	18.2	40.2	26.5
Extubating Day		POD#2	POD#1	POD#6	POD#9	POD#6
TPN		No	Yes	Yes	Yes	Yes
TPN duration		N/A	4 days (POD#1–4)	5 days (POD#3–7)	10 days (POD#1–10)	15 days (POD#1–15)
Highest TPN Protein Dose (g/kg/day)		N/A	0.78	1.00	1.00	1.34
Insulin use		Yes	Yes	Yes	Yes	Yes
Albumin infusion		Yes	Yes	Yes	Yes	Yes
Albumin dose		1.25 g/kg × 1 day	0.5 g/kg × 4 days	0.5 g/kg × 2 days, 1.0 g/kg × 5 days	0.5 g/kg × 2 days, 1.0 g/kg × 1 day	0.5 g/kg × 3 days, 1.0 g/kg × 1 day
Enteral feeding initiation		POD#3	POD#3	POD#6	POD#2 failed. POD#10	POD#7 failed. POD#14
Feeding complications		No	No	No	Feeding intolerance with vomiting	Feeding intolerance with vomiting
Post-op inpatient nutrition protein goal (g/kg/day)		1.5–2.0	1.0–1.5	1.0–1.5	1.2–2.0	1.5
Stable protein dose achievement		POD#4	POD#5	POD#14	POD#12	POD#12
Miscellaneous		Maximum protein intake as high as 1.6 g/kg/day during admission for transplant		Protein increased additionally after discharge		Protein goal was decreased due to worsening renal dysfunction 3 months after transplant

POD; post-operative day, TPN; total parenteral nutrition, N/A; not assessed.
